# When cues take the wheel: cue-control across alcohol use disorder, binge-eating disorder, gambling disorder, and internet gaming disorder

**DOI:** 10.3389/fpsyg.2026.1778384

**Published:** 2026-04-10

**Authors:** Erik L. Bode, Samanda Krasniqi, Annika Rosenthal, Eva Friedel, Florian Schlagenhauf, Miriam Sebold

**Affiliations:** 1 Department of Psychiatry and Neurosciences, Charité - Universitätsmedizin Berlin, Corporate Member of Freie Universität Berlin, Humboldt-Universität zu Berlin, Berlin, Germany; 2 Charité—Universitätsmedizin Berlin, Corporate Member of Freie Universität Berlin and Humboldt-Universität zu Berlin, Einstein Center for Neurosciences Berlin, Berlin, Germany; 3 Department of Psychology, Faculty of Life Sciences, Humboldt-Universität zu Berlin, Berlin, Germany; 4 Faculty of Economics and Law, Technische Hochschule Aschaffenburg—University of Applied Sciences, Aschaffenburg, Germany

**Keywords:** alcohol use disorder, binge eating disorder, cue-reactivity, gambling disorder, incentive salience, internet gaming disorder, Pavlovian-to-instrumental transfer

## Abstract

Cues associated with rewarding outcomes can strongly influence behavior, shaping attention, motivation, and action selection. Across escalating and compulsive behaviors such as alcohol use disorder (AUD), binge-eating disorder (BED), and gambling disorder (GD), as well as the related case of internet gaming disorder (IGD), cue-triggered responses are commonly observed, yet their underlying behavioral mechanisms remain heterogeneous and incompletely understood. This review synthesizes evidence from experimental paradigms that probe cue-driven control over behavior, with a particular focus on Pavlovian conditioning, Pavlovian-to-Instrumental Transfer (PIT), and related Pavlovian-to-decision paradigms in humans and animals. We highlight how Pavlovian cues can invigorate instrumental responding, bias choice among available actions, or capture attention independently of current goals, and how these effects vary across reinforcer type, task structure, and individual differences. Rather than supporting a unitary cue-reactivity account, the literature points to partially overlapping processes, including incentive salience attribution, attentional capture, and Pavlovian modulation of instrumental control, that appear across disorders but are differentially expressed across subgroups. We argue that paradigms that assess cue-control provide a valuable mechanistic bridge between basic learning theory and clinically relevant behavior, while also posing methodological challenges for interpretation and translation. Understanding when and how cues gain control over action may inform more targeted, mechanism-based interventions for excessive behaviors.

## Introduction

Cue-reactivity refers to the phenomenon that stimuli previously associated with reinforcers can elicit conditioned physiological and motivational responses. Consistent with this, exposure to such cues can induce strong motivational states and increase difficulties in resisting engagement. In some individuals, the motivational pull of such cues becomes strong enough to impair behavioral control, as seen in escalating behaviors such as alcohol use disorder (AUD), binge-eating disorder (BED), and gambling disorder (GD). Reflecting these parallels, the DSM-5 groups AUD and GD under the joint category of addictive disorders [American Psychiatric Association (APA), 2013]. Excessive overeating, including forms observed in BED, shares notable features with addictive behaviors (Meule and Gearhardt, 2014), yet remains classified under eating disorders, potentially obscuring mechanistic overlaps with addiction phenotypes. Moreover, similar behavioral patterns are observed in internet gaming disorder (IGD), which is included in the DSM-5 as a condition for further study rather than an established clinical diagnosis. Distinct from gambling disorder, it provides a conceptually informative case for understanding cue-driven motivational processes [American Psychiatric Association (APA), 2013]. Despite these distinct diagnostic labels, these conditions show striking behavioral similarities, characterized by heightened cue-reactivity that can undermine self-regulatory processes and result in recurrent loss of control and powerful cue-triggered urges toward alcohol, palatable food, gambling, or gaming outcomes. Accordingly, we focus on AUD, BED, and GD as clinically established conditions with shared clinical features, while differing in reinforcer type and reward context, spanning substance-based, behavioral, and natural reward domains. IGD is included as a related boundary case that permits examination of cue-driven modulation in a non-pharmacological, skill-based reward context.

A dimensional, mechanistic perspective helps contextualize these phenotypic overlaps. Individuals meeting identical diagnostic criteria can differ markedly in their cue-triggered motivation. For example, two individuals with AUD may differ in whether they experience cue-triggered urges (Sebold et al., 2022). Similar heterogeneity is reported in BED and GD (Kim and King, 2020; Meule and Gearhardt, 2014). These observations indicate that motivational and behavioral control processes vary substantially both within and across diagnostic categories. Such variation may help explain why uniform diagnostic labels often fail to capture the mechanisms that drive excessive engagement. Within the literature, craving is typically defined as a motivational state that promotes engagement in substance use and is formalized as a diagnostic criterion for substance use disorders (Sayette, 2016). Across substances, gambling, and food, craving is typically described as an intense desire or urge to consume or engage, accompanied by intrusive cognitions and a sense of reduced control (Drummond, 2001; Miele et al., 2023). Craving is frequently triggered by conditioned cues and perceived opportunities to consume (Sayette, 2016), and recent psychometric work shows that craving measures across substances, gambling, and eating cluster on shared latent components reflecting urge intensity, cue-elicited desire, intrusive thoughts, and perceived loss of control, rather than disorder-specific factors (Miele et al., 2023). Together, this supports the view of craving as a transdiagnostic motivational construct relevant to multiple excessive behaviors.

Experimental cue-reactivity paradigms provide a crucial access path to understanding these processes. Disorder-relevant cues such as alcohol images, gambling scenes, or palatable foods reliably increase subjective craving and attentional engagement compared to neutral stimuli (Carter and Tiffany, 1999). In AUD, alcohol cues consistently evoke heightened craving and attentional bias (Hanlon et al., 2018; Heinz et al., 2004). Palatable food cues show similar effects in BED (Kenny, 2011; Schienle et al., 2009), and gambling cues elicit craving and physiological arousal in GD (Clark, 2014; Limbrick-Oldfield et al., 2017). Importantly, although most cue-reactivity studies use disorder-specific stimuli, cue-triggered motivational processes often can generalize beyond symptom-specific cues, suggesting that cue-driven modulation of action may reflect a broader, cross-diagnostic mechanism. This generalization likely reflects shared learning and motivational mechanisms, even though the specific reinforcers and contexts through which these mechanisms are expressed differ across disorders. At the same time, cue-reactivity designs largely capture affective and physiological responses and tell us little about how cues change ongoing choice, effort, or behavior.

The incentive-sensitization framework offers one influential account of how cues gain such motivational impact. Repeated reinforcement in substance use disorders is proposed to sensitize reward-related systems, causing cues to become attention-grabbing and capable of driving behavior even when the underlying reward is not strongly “liked” (Berridge and Robinson, 1993, 2016). Such processes have also been suggested for palatable food and gambling cues (Clark, 2014; Kenny, 2011). Cue-triggered urges are therefore clinically meaningful, often preceding episodes of loss of control across AUD, BED, and GD (Kim and King, 2020; Meule and Gearhardt, 2014).

Despite shared features, AUD, BED, and GD differ in the specific reinforcers that drive behavior and in the contexts in which cues operate. AUD and BED involve primary reinforcers such as alcohol and food, which have innate hedonic value and are closely tied to interoceptive and metabolic states, and whose associated cues may become linked to bodily states and stress regulation (Kenny, 2011; Koob and Volkow, 2016; Sescousse et al., 2013). GD, in contrast, centers exclusively on secondary reinforcers—particularly money—whose value depends on learned associations and abstract beliefs about probability and reward (Clark, 2014; Sescousse et al., 2013). Gambling environments contain dense, salient audiovisual cues that exploit learning biases such as near-miss effects and illusions of control (Clark, 2014). Despite these differences, converging imaging evidence indicates that alcohol, food, and gambling cues engage overlapping motivational neural circuits (Sescousse et al., 2013). Moreover, repeated overconsumption can blunt responsiveness to natural rewards while amplifying cue-driven motivation, particularly in obesity and BED (Kenny, 2011; Volkow et al., 2019).

Internet Gaming Disorder (IGD) represents a boundary case for examining cue-driven modulation of behavior in the absence of direct pharmacological or metabolic reinforcement, thereby placing greater emphasis on learned cue-action associations within complex, skill-based environments. IGD is defined in the DSM-5 as a pattern of persistent and recurrent gaming associated with clinically significant impairment, with diagnosis suggested when at least five of nine criteria are met within a 12-month period. These criteria reflect core domains common to other addictive disorders, including impaired control over engagement with gaming, persistence despite negative consequences, and addiction-related features such as tolerance and withdrawal [American Psychiatric Association (APA), 2013]. Although gaming does not involve a direct pharmacological or metabolic reinforcer, it relies on variable-ratio reward schedules, symbolic gains and losses, and persistent learned cues, closely paralleling motivational features of gambling (Clark, 2014). At the same time, skill and performance contribute substantially to reinforcement, making IGD a useful case for testing how cue-driven modulation of behavior manifests in complex, learned action environments.

While cue-reactivity paradigms have been central for characterizing responses to disorder-relevant stimuli, they do not quantify how cues actively shape instrumental choice and behavior. Moreover, craving does not always translate into actual engagement, and individuals may act on cues even in the absence of strong subjective desire (Drummond, 2001; Sayette, 2016). Craving describes a subjective motivational state, whereas conditioned cues can exert a causal influence on instrumental behavior even in the absence of conscious desire. In this review, we therefore focus on cue-control across alcohol use disorder (AUD), binge-eating disorder (BED), gambling disorder (GD), and the related case of internet gaming disorder (IGD), and define cue-control as the set of processes by which reward-associated cues shape motivation, decision-making, and behavior, including how cues bias choice and action selection. Cue-control encompasses subjective and physiological cue-reactivity, as well as the extent to which conditioned stimuli can energize, bias, or disrupt ongoing behavior. Paradigms targeting cue-control, most prominently Pavlovian-to-Instrumental Transfer (PIT), directly quantify how Pavlovian cues invigorate or bias instrumental actions, making them particularly well suited to operationalize cue-control. Preclinical studies demonstrate robust PIT effects for drug- and food-paired cues (Corbit and Balleine, 2011; Everitt and Robbins, 2016), and translational work in AUD indicates enhanced PIT even with non-drug reinforcers (Doñamayor et al., 2021). More broadly, learned cues have been shown to bias value-based decision-making and risk-taking.

**Figure 1:**
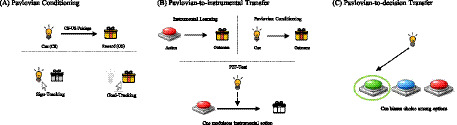
Conceptual overview of translational cue driven paradigms. **(A)** Pavlovian conditioning. A neutral cue (conditioned stimulus, CS) is repeatedly paired with a reward (unconditioned stimulus, US), allowing the cue to acquire predictive value. In rodents, sign-tracking and goal-tracking are not assessed in a discrete test phase but emerge during acquisition, with sign-trackers directing conditioned approach toward the CS itself and goal-trackers approaching the reward location, despite identical CS–US learning histories. **(B)** Pavlovian-to-instrumental transfer (PIT). Instrumental learning (action → outcome) and Pavlovian conditioning (cue → outcome) are acquired separately. During the PIT test, presentation of the Pavlovian cue modulates ongoing instrumental responding, reflecting cue induced invigoration or biasing of action. **(C)** Pavlovian-to-decision transfer. A Pavlovian cue presented at choice biases selection among multiple response options, illustrating how reward predictive cues can guide decisions even when the cue is not informative about the optimal action.

Together, these observations motivate an integrative examination of cue-control as a transdiagnostic yet disorder-sensitive mechanism in AUD, BED, and GD and the selective inclusion of IGD. In this narrative review, we selectively synthesize experimental paradigms that directly assess how reward-associated cues modulate instrumental action and decision-making across disorders. We integrate animal and human evidence across AUD, BED, GD, and the related case of IGD, with a primary emphasis on Pavlovian and Pavlovian-to-Instrumental Transfer (PIT) paradigms and a secondary focus on Pavlovian-to-decision paradigms (Figures 1A–C). Here, we use the term Pavlovian-to-decision paradigms to refer to tasks in which reward-associated cues bias value-based choice or decision policies, regardless of whether cue-outcome associations are acquired through an explicit Pavlovian conditioning phase or through repeated cue-choice pairings. By emphasizing behavioral readouts and outlining neural mechanisms where informative, we aim to clarify the mechanisms by which reward-associated cues bias instrumental behavior and decision-making across addictive and overeating phenotypes. In doing so, we conceptualize cue-control as a transdiagnostic framework that helps situate craving and loss of control within shared learning and motivational processes.

### Literature identification and scope

This review follows a narrative, theory-driven approach aimed at synthesizing mechanistic insights into cue-driven modulation of behavior and decision-making across diagnostic categories. Literature was identified through targeted database searches (e.g., PubMed, PsycINFO) using combinations of keywords including “Pavlovian-to-instrumental transfer,” “cue-reactivity,” “approach bias,” “sign-tracking,” and relevant disorder terms (e.g., alcohol use disorder, binge-eating disorder, overeating, gambling disorder, internet gaming disorder). We prioritized empirical studies employing experimental paradigms that directly assessed cue-driven modulation of instrumental behavior or decision-making in both humans and animals, with particular emphasis on translational alignment between species. Study selection was guided by conceptual relevance rather than exhaustiveness, with a primary focus on research from the past two decades, supplemented by seminal earlier work and studies identified through citation tracking.

## Pavlovian conditioning: sign-tracking vs. goal-tracking

One experimental framework that has been particularly informative for understanding how reward-associated cues acquire motivational control over behavior is Pavlovian conditioning. Pavlovian conditioning procedures, often termed autoshaping or Pavlovian conditioned approach (PCA), pair a neutral cue (CS) with a rewarding unconditioned stimulus (US), such that the cue gains behavioral relevance and elicits conditioned responding independently of instrumental action (Figure 1A) (Flagel et al., 2009). From a cue-control perspective, PCA is informative because it distinguishes between a cue predicting reward and the cue itself becoming motivationally powerful (Robinson and Flagel, 2009). This distinction is central for understanding when reward-associated cues exert disproportionate control over behavior, particularly in disorder-relevant contexts.

In standard PCA procedures, animals receive response-independent CS-US pairings, for example presentation of a lever followed by reward delivery (Fitzpatrick and Morrow, 2016; Robinson and Flagel, 2009). With training, distinct conditioned response patterns emerge, with sign-trackers (STs) approaching and engaging the cue itself and goal-trackers (GTs) directing behavior toward the reward location during the CS period. Although both groups learn the predictive relationship (Robinson and Flagel, 2009), the cue acquires greater motivational value in STs, functioning as a stronger conditioned reinforcer than in GTs (Flagel et al., 2009; Robinson and Flagel, 2009). Predictive learning alone therefore does not determine whether a cue becomes motivationally potent.

Crucially, ST and GT are best understood as positions along a continuum rather than discrete types. Most laboratories classify animals using a composite PCA index based on lever- and magazine-directed measures (i.e., approach toward the CS versus the reward location) (Fitzpatrick and Morrow, 2016; Meyer et al., 2012). Based on researcher-defined cutoffs along this continuous index, animals are typically categorized as sign-trackers, goal-trackers, or intermediates. Because the underlying distribution is continuous, phenotype assignment depends on researcher-chosen cutoffs, which vary across paradigms and laboratories (Fitzpatrick and Morrow, 2016; Meyer et al., 2012; Robinson and Flagel, 2009). Accordingly, ST and GT labels reflect operational thresholds imposed on graded variation rather than natural categories, limiting simple categorical interpretations and requiring caution when translating findings across paradigms or reinforcers.

Beyond classification, a trait versus state perspective is critical for translational interpretation. The PCA literature frames ST and GT as behavioral phenotypes indexing a propensity to attribute incentive salience to reward-predictive cues (Flagel et al., 2009; Meyer et al., 2012; Sarter and Phillips, 2018), with evidence supporting trait-like stability and predictive validity across tasks (Meyer et al., 2012). At the same time, expression of sign- versus goal-tracking is shaped by task and contextual features, including cue properties and training parameters (Fitzpatrick and Morrow, 2016; Meyer et al., 2012; Sarter and Phillips, 2018). Under some conditions, animals may sign-track to one cue yet goal-track to another, indicating that cue-reactivity can vary across reinforcers and contexts (Meyer et al., 2014; Sarter and Phillips, 2018). This state–trait interplay has direct relevance for disorder-focused questions because it raises whether heightened cue-reactivity generalizes across reward domains or emerges selectively under specific reinforcer and contextual conditions.

Addressing these questions in humans is methodologically challenging because human Pavlovian tasks rarely elicit overt cue-directed approach, making direct classification of sign-tracking difficult. While a child-friendly autoshaping analogue demonstrated that children classified as sign-trackers exhibited higher impulsivity and poorer inhibitory control (Colaizzi et al., 2023; Joyner et al., 2018),direct autoshaping procedures often evoke limited behavioral differentiation in adults (Colaizzi et al., 2020; Wardle et al., 2018). Thus, several recent studies have turned to more subtle behavioral correlates of the ST/GT continuum, such as stimulus-locked eye-gaze patterns (Cope et al., 2023; Dinu et al., 2024; Heck et al., 2025; Schad et al., 2019). Consistent with animal work, individuals whose gazes are more strongly captured by reward-predictive cues show higher trait impulsivity (Garofalo and Di Pellegrino, 2015) and stronger neural signatures of rigid and inflexible learning (Gläscher et al., 2010; Schad et al., 2019). While such associations link sign-tracking tendencies to impulsivity-related behaviors, the ST/GT framework focuses more specifically on the learning processes through which reward-predictive cues acquire motivational control over behavior.

Because human Pavlovian conditioning tasks often fail to elicit robust cue-directed behavior, several researchers have turned to alternative paradigms to infer sign- and goal-tracking tendencies. One prominent paradigm involves *value-modulated attentional capture* (VMAC), in which high-value distractors compete for attention (Albertella et al., 2019b; Garre-Frutos et al., 2025). Similar to animal work, these tasks reveal that some individuals display pronounced cue-driven attentional capture that is difficult to suppress (Anderson et al., 2013; Anderson and Yantis, 2013), and this variability relates to working memory capacity (Watson et al., 2019) and compulsivity (Albertella et al., 2019b). These paradigms therefore provide an indirect but quantifiable index of cue-control, capturing the extent to which reward-associated stimuli bias attention and interfere with behavior even when task-irrelevant. Other studies have used indices of reward-seeking behavior or attentional bias toward motivationally salient stimuli to capture related constructs (Field and Cox, 2008; Jasinska et al., 2014; Saunders and Robinson, 2013). Although these paradigms do not replicate the exact behavioral phenotype observed in rodent autoshaping, they tap into individual differences in cue-reactivity that are conceptually adjacent to the sign-tracking/goal-tracking continuum.

Together, these approaches illustrate cross-species variability in cue-reactivity while highlighting the methodological challenges of translating Pavlovian phenotypes to humans. In rodents, sign-trackers come to treat reward-predictive cues as motivationally attractive and will work to obtain them, whereas goal-trackers preferentially orient toward the site of reward delivery despite equivalent knowledge of the cue-outcome relationship (Flagel et al., 2011). Importantly, sign- and goal-tracking reflect positions along a continuum of conditioned responding rather than discrete categories, with many individuals exhibiting intermediate phenotypes. Parallel work in humans suggests comparable individual differences in cue-reactivity, with sign-tracking-like individuals showing stronger cue-driven behavioral and attentional capture, whereas goal-tracking-like individuals rely more strongly on predictive relationships between cues and outcomes (Schad et al., 2020). However, because overt cue-directed approach is rarely expressed in humans, these phenotypes must typically be inferred from indirect behavioral markers such as gaze allocation or attentional capture. Together, these findings illustrate how Pavlovian cue-control paradigms provide a translational framework linking basic learning mechanisms with variability in cue-driven behavior across species while also highlighting the methodological challenges of translating these processes to human studies.

### Pavlovian conditioning in alcohol use disorder

Research on alcohol-related cue-reactivity within Pavlovian conditioning frameworks addresses two complementary questions. The first is whether alcohol exposure promotes sign-tracking toward alcohol cues, and the second is whether sign-tracking phenotypes themselves confer vulnerability to problematic alcohol use. In animal models, the first line of work asks whether alcohol exposure shifts behavior toward sign-tracking. Across several studies, repeated pairings of alcohol-predictive cues increase cue-directed approach behavior, indicating a gradual shift from goal-tracking to sign-tracking with training (Srey et al., 2015; Tomie and Sharma, 2014). Related work shows that alcohol-paired cues can elicit strong sign-tracking even when later paired with non-alcoholic rewards (Tomie, 2002). Reported effects vary depending on training procedures and ethanol administration protocols (Versaggi et al., 2016).

A second line of work examines whether sign-tracking itself reflects vulnerability to problematic alcohol use. Locomotor response to novelty—a known predictor of addiction vulnerability—is strongly associated with sign-tracking tendencies (Flagel et al., 2009), suggesting shared underlying traits. Sign-trackers exhibit stronger conditioned reinforcement of alcohol cues (Srey et al., 2015), heightened ethanol-induced impulsivity (Tomie et al., 1998a), and increased voluntary ethanol intake (Tomie, 2002). Learning history modulates these effects, as adolescent autoshaping enhances later alcohol consumption (Anderson and Spear, 2011). Yet findings are not uniform: alcohol-preferring rat lines do not consistently show elevated sign-tracking (Peña-Oliver et al., 2015), and males appear more prone to sign-tracking than females (Barker and Taylor, 2019), indicating heterogeneity in vulnerability pathways.

Turning to humans, studies using eye-gaze, attentional capture, and conditioning paradigms reveal broadly similar links between alcohol use and cue-reactivity, although these effects show greater methodological variability. Individuals showing stronger stimulus-locked gaze patterns report higher alcohol use in subclinical samples (Heck et al., 2025). In VMAC tasks—where attentional capture by reward cues is used as a behavioral marker of sign-tracking—greater capture by high-value distractors correlates with risky alcohol use (Albertella et al., 2019b), alcohol-related problems (Albertella et al., 2019a), and alcohol drinking severity (Watson et al., 2024), although one gamified VMAC study did not replicate these associations (Freichel et al., 2024). Conditioning studies further show that neutral cues paired with alcohol elicit attentional biases proportional to alcohol liking (Mayo and De Wit, 2016), and heavy drinkers develop contextual preferences for alcohol-paired environments without explicit contingency awareness (Childs and De Wit, 2016).

Taken together, across animal and human studies, alcohol exposure can enhance cue-driven responding and heightened cue-reactivity may index vulnerability to problematic alcohol use, although findings remain heterogeneous. Consistent with the state–trait framework outlined earlier, current findings leave open whether heightened cue-reactivity reflects a relatively stable vulnerability marker, a context-dependent expression shaped by alcohol exposure, or an interaction of both. Further longitudinal and mechanistically aligned work will be needed to clarify whether heightened cue-driven behavior reflects a vulnerability factor, a consequence of alcohol use, or both.

### Pavlovian conditioning in pathological gambling

To date, relatively few studies have examined the sign-tracking/goal-tracking continuum in the context of gambling disorder, particularly within the animal literature. Novel rodent models such as gambling tasks and slot-machine analogues, which vary reward magnitude and risk, suggest that sign-tracking may be linked to suboptimal, high-risk decision-making (Winstanley and Clark, 2015). Rats showing stronger sign-tracking choose high-risk/high-reward options more often and make more premature responses (Swintosky et al., 2021). Given established links between risk preference, novelty seeking (Rivalan et al., 2009), and gambling vulnerability (Hales et al., 2025), these findings suggest that variation in cue-reactivity may contribute to gambling-relevant choice patterns. Moreover, recent research indicates that reward uncertainty—a hallmark of gambling—plays a critical role in shaping sign-tracking behavior. Higher levels of reward uncertainty, as observed in slot-machines, enhance both the propensity and intensity with which animals develop sign-tracking phenotypes, enhancing cue-driven behavioral tendencies and increasing the motivational pull of reward-predictive cues (Anselme and Güntürkün, 2019; Anselme and Robinson, 2013; Robinson et al., 2014, 2019). In experimental paradigms, such uncertainty is often implemented through variable reinforcement schedules or variable inter-trial intervals, in which reward delivery occurs unpredictably rather than at fixed times. These conditioning parameters can influence the emergence and expression of sign- and goal-tracking behavior (Fitzpatrick and Morrow, 2016). Consistent with this, anxiety-related traits modulate cue-reactivity in gambling-relevant contexts, with experimental data indicating that reward uncertainty selectively enhances sign-tracking behavior in high-anxiety animals (Hellberg et al., 2018).

In humans, converging evidence indicates that win-related audiovisual cues reliably capture attention and increase motivational engagement, even when they are task-irrelevant. Casino-style cues draw visual attention and interfere with ongoing task performance in healthy participants (Cherkasova et al., 2018; Spetch et al., 2020), consistent with sign-tracking-like attentional capture by reward-predictive stimuli. Similar effects are observed in VMAC paradigms, where reward-paired sensory feedback enhances attentional capture despite being irrelevant to task goals (Pearson et al., 2024). Vulnerability factors further shape these cue-reactivity effects: individuals with higher anxiety, stress, depressive symptoms, or gambling severity show stronger attentional and motivational responses to reward-paired cues during simulated slot-machine play (Arshad et al., 2025).

Across animal models and human experimental paradigms, findings suggest that gambling-related cues, particularly under conditions of reward uncertainty, readily acquire incentive salience and exert strong influence over attention and motivational engagement. Importantly, many gambling environments operate under variable reinforcement schedules, in which rewards occur unpredictably rather than after a fixed number of responses. Such schedules increase reward uncertainty, and experimental work indicates that similar uncertainty enhances the emergence and intensity of sign-tracking phenotypes in animal models (Anselme and Robinson, 2013; Fitzpatrick and Morrow, 2016; Robinson et al., 2014, 2019). Consistent with this, the strong modulation by reward uncertainty and anxiety-related traits aligns with a state-sensitive expression of cue-reactivity, rather than a uniform domain-general vulnerability.

### Pavlovian conditioning in binge-eating and obesity

Animal studies examining sign-tracking and overeating show mixed but informative patterns. Several lines of evidence suggest that sign-tracking may function as a vulnerability marker for obesity and binge-like intake, reflecting heightened cue-driven motivational control. Rats that later develop obesity already display heightened conditioned approach behavior before any weight gain occurs (Robinson et al., 2015), and food-predictive cues reinstate food seeking more strongly in sign-trackers than in goal-trackers, with hunger amplifying this effect only in sign-trackers (Yager and Robinson, 2010). These findings indicate that individuals who attribute high incentive-salience to food cues may be particularly susceptible to cue-induced overeating. However, not all data align with this account, as some studies report no systematic relationship between obesity vulnerability and cue-reactivity (Jarosz et al., 2007), and obesity-resistant rats have shown stronger cocaine conditioned place preference than obesity-prone rats (Thanos et al., 2010), challenging the notion of a generalized incentive-salience bias in obesity-prone phenotypes.

Cue-reactive changes can also emerge from excessive food intake. In animal models, highly palatable diets do not uniformly boost motivation but selectively amplify cue-reactivity in certain subgroups. For instance, only rats that developed obesity on a “junk-food” diet showed a significantly higher willingness to work for a reward-predictive cue (Robinson et al., 2015). Similarly, binge-like eating has been associated with stronger conditioned place preference for food-paired cues relative to controls (Velázquez-Sánchez et al., 2015), and body fat has been shown to correlate with preference for food-associated environments in hamsters (Moran and Delville, 2024).

However, more recent evidence challenges the idea that excessive intake uniformly enhances incentive salience attribution. Rats with binge-like sugar consumption have been reported to show reduced conditioned place preference for sucrose-paired environments, despite intact preference for cocaine-paired contexts (Smail-Crevier et al., 2018). This dissociation suggests that binge-like sugar intake may selectively disrupt reward processing for sucrose, rather than producing a generalized alteration across reward types.

In humans, emerging work has examined whether sign- and goal-tracking-like tendencies can be detected using neural indices of cue-reactivity. Using EEG, Versace et al. (2016) classified individuals based on the relative magnitude of late positive potential responses to food cues compared with other emotionally arousing stimuli. An ST-like profile, characterized by heightened neural responses to food cues alongside attenuated responses to other pleasant stimuli, was more prevalent among individuals with obesity, although lean and obese participants were present in each group. Extending this work, individuals showing larger neural responses to food cues relative to other positive stimuli were more susceptible to cue-induced eating, independent of BMI (Versace et al., 2019). These findings parallel animal findings in highlighting endophenotypic differences in the degree to which food cues acquire motivational significance.

Taken together, across species, variation in cue-reactivity appears relevant for overeating, but findings remain mixed and are shaped by developmental stage, diet exposure, and reward specificity. Overall, findings suggest that cue-reactivity in overeating is shaped by both predispositional differences and reinforcement history, and it remains unresolved whether observed subgroup differences precede excessive intake or emerge as a consequence. While neural and behavioral evidence in humans mirrors animal work in identifying subgroups that assign greater motivational value to food cues, it remains unresolved whether these differences precede excessive intake or emerge as a consequence of it.

## Pavlovian-to-instrumental transfer and Pavlovian-to-decision transfer

Findings from sign-tracking paradigms highlight individual differences in how strongly cues attract attention and motivate approach, but they do not directly address how such cues influence instrumental choice and action. The Pavlovian-to-Instrumental Transfer (PIT) paradigm fills this gap by assessing the extent to which Pavlovian cues invigorate, or bias independently learned actions. In PIT, a conditioned stimulus that predicts a reward potentiates an instrumental response trained in a separate context (Dickinson et al., 2000), providing a behavioral index of how conditioned environmental cues motivate and shape ongoing behavior (Figure 1B). Although PIT is often illustrated using canonical associative-learning tasks, the empirical literature encompasses a broad range of PIT and Pavlovian-to-decision paradigms (Figure 1C), ranging from classical three-phase PIT designs to gambling-like choice procedures and cue-based decision-making tasks (Cartoni et al., 2016). Despite substantial differences in task structure, these paradigms share the core functional feature that reward-associated Pavlovian cues bias or modulate instrumental behavior or decision policies, providing a unifying framework for interpreting cross-species and cross-disorder findings within a cue-control perspective.

PIT effects are commonly divided into general and specific forms (Ostlund and Balleine, 2007). In general PIT, reward-predictive cues broadly invigorate instrumental responding, even for actions that earn different outcomes. In specific PIT, cues selectively bias actions linked to the same outcome they predict. Across PIT variants, cue-driven influences are highly sensitive to dopaminergic modulation. Systemic dopamine antagonists reliably reduce PIT expression (Dickinson et al., 2000), and cues associated with reward uncertainty amplify phasic dopamine firing (Fiorillo et al., 2003), enhancing their motivational impact.

These cue-driven influences can override flexible adjustment even when instrumental contingencies remain stable. Pavlovian cues invigorate reward seeking in ways that can conflict with outcome value or current motivational state, and stronger PIT effects are associated with addiction-like behaviors in rodents, including escalated drug seeking and compulsive responding (Barker et al., 2012). Related developmental work shows that adolescents display heightened cue-triggered reward seeking with reduced updating of outcome expectancy (Marshall et al., 2020), and PIT-like phenomena also emerge in aversive contexts where threat-paired cues potentiate avoidance responding (Campese et al., 2013).

While these findings highlight PIT’s translational potential, several challenges remain. Human paradigms vary in ecological validity, and PIT performance depends on the integrity of both Pavlovian and instrumental learning phases, which can complicate interpretation in clinical samples (Garbusow et al., 2014). Nonetheless, PIT offers a mechanistic bridge between basic associative learning processes and real-world contexts in which cues shape ongoing behavior. By capturing how Pavlovian signals modulate instrumental responding, PIT provides a framework for examining cue-driven motivational biases across domains and sets the stage for understanding their potential relevance in alcohol, overeating, internet gaming and gambling -related conditions.

### Pavlovian-to-instrumental transfer and Pavlovian-to-decision transfer in alcohol use disorder

Preclinical work provides converging evidence that alcohol-predictive cues can invigorate instrumental responding through Pavlovian-to-instrumental transfer mechanisms. In classic three-phase PIT paradigms, cues paired with ethanol selectively increase lever pressing for ethanol under extinction, demonstrating outcome-specific PIT (Corbit et al., 2012). Related PIT-like designs similarly show that ethanol-paired cues enhance operant responding compared to neutral cues, even when instrumental contingencies remain unchanged (Troisi, 2006). Together, these findings indicate that Pavlovian alcohol cues can selectively bias alcohol-seeking actions, primarily through largely outcome-specific PIT effects consistent with incentive-motivational transfer rather than simple stimulus control.

As outlined in the preceding autoshaping section, ethanol-predictive cues readily acquire incentive salience through Pavlovian conditioning and can invigorate approach and consumption even outside instrumental contingencies (Krank, 2003; Tomie et al., 2003). Importantly, ethanol exposure itself can bias the form of Pavlovian responding, increasing the propensity to attribute incentive salience to cues, for example by shifting the balance between sign-tracking and goal-tracking (Tomie et al., 1998b). These findings are critical for interpreting PIT effects in alcohol use, as PIT necessarily builds on the motivational properties acquired during Pavlovian learning rather than reflecting a *de novo* process.

Individual differences further modulate the strength of cue-driven alcohol seeking. Animals with stronger sign-tracking tendencies show greater cue-induced reinstatement, whereas ethanol exposure modifies conditioned responding without consistently increasing sign-tracking across individuals (Versaggi et al., 2016). These findings suggest that individual differences may differentially modulate general versus outcome-specific PIT components. This pattern supports a dual interpretation of PIT-related effects in alcohol use, reflecting both a vulnerability factor linked to trait-like cue-reactivity and a consequence of alcohol exposure that further amplifies cue driven modulation over behavior.

In humans, evidence for PIT-related cue effects in alcohol use disorder is more variable, reflecting substantial methodological heterogeneity across paradigms. In formal translational PIT tasks, alcohol-predictive cues selectively enhance responses for alcohol-associated actions in detoxified individuals with AUD, indicating largely intact outcome-specific PIT rather than exaggerated cue-driven responding (Garbusow et al., 2014). Notably, neuroimaging work shows that PIT-related activity in the nucleus accumbens predicts relapse risk, linking cue-triggered motivational transfer to clinically relevant outcomes even in the absence of behavioral group differences (Garbusow et al., 2016). In contrast, studies using modified PIT designs report that broader cue-induced response invigoration (reflecting general PIT rather than specific PIT), including to non-alcohol cues, is associated with impulsivity and relapse vulnerability (Sommer et al., 2017, 2020), suggesting that heightened general cue-reactivity may be more relevant than alcohol-specific PIT effects per se.

More recent developmental and longitudinal work indicates that greater PIT-like susceptibility predicts hazardous drinking trajectories in young adults (Chen et al., 2021, 2023). Converging evidence shows that individuals exhibiting stronger PIT effects are also more likely to show neural patterns associated with cue-driven motivational transfer that predict drinking behavior and relapse risk (Sekutowicz et al., 2019), supporting the view that PIT-related processes index vulnerability rather than reflecting a uniform loss of flexible, outcome-sensitive action control. Consistent with this interpretation, outcome-specific PIT appears largely preserved in AUD, with no evidence for a global impairment in the ability to adjust behavior based on current outcome values (Giannone et al., 2024; Van Timmeren et al., 2020).

Taken together, animal and human studies suggest that alcohol-predictive cues can selectively invigorate instrumental alcohol seeking through PIT mechanisms, although the strength and expression of these effects vary substantially across paradigms and levels of analysis. Rather than reflecting a categorical deficit in instrumental control, PIT-related effects in AUD appear to indicate increased susceptibility to cue-driven motivational capture, operating alongside largely intact outcome-sensitive and flexible action control.

### Pavlovian-to-instrumental transfer and Pavlovian-to-decision transfer in pathological gambling and gaming

Gambling environments are saturated with reward-paired cues such as lights and sounds, which closely resemble Pavlovian conditioned stimuli and can bias instrumental responding even when contingencies remain stable. Rodent models have leveraged this by adding audiovisual win cues to the rat gambling task (rGT) (Zeeb et al., 2009), effectively transforming it into a PIT-like paradigm in which Pavlovian signals modulate gambling-like choice. Across studies, these win-paired cues reliably shift behavior toward high-risk, high-reward options and reduced sensitivity to punishment (Barrus et al., 2015). They also increase impulsive behavior, reflected in elevated premature responding (Ferland et al., 2019; Hrelja et al., 2025), indicating that salient reward cues can simultaneously bias valuation and invigorate action.

Beyond these group-level effects, substantial individual differences shape the magnitude of cue-driven biases. Animals with a baseline preference for risky options show amplified cue-induced destabilization of choice (Hrelja et al., 2025). While adolescent rats are inherently more risk-seeking than adults, they do not exhibit cue-induced increases in risky choice. This suggests that the “transfer of salience”—the process by which a cue gains the power to drive behavior—is developmentally limited during adolescence (Zeng et al., 2023). Chronic exposure to cued gambling schedules can further exacerbate vulnerability, with extended cue training increasing susceptibility to addiction-like behaviors in some models (Ferland et al., 2019).

A complementary paradigm, the suboptimal choice procedure (SCP), demonstrates that cue salience can override reinforcement contingencies. When high-salience cues signal the low-probability option, rats, particularly sign-trackers, shift toward this disadvantageous choice (Orduña and Alba, 2020). When cue salience is equated, rats behave optimally. Although SCP effects reflect conditioned reinforcement more than PIT per se, both paradigms converge on the conclusion that incentive-salient cues can dominate decision strategies, paralleling gambling-like distortions in humans. Together, these findings suggest that reward-paired cues can systematically bias gambling-like behavior in rodents, particularly in phenotypes marked by heightened incentive salience, although this effect does not reliably emerge in adolescents.

Similarly, human research has relied on Pavlovian-to-decision transfer approaches to examine how gambling cues bias instrumental choice under stable contingencies. Across studies, gambling-related images or affective cues reliably increase risky decisions, especially in individuals with gambling disorder (Genauck et al., 2020, 2021). Computational modeling and multivariate fMRI analyses indicate that these effects reflect cue-induced shifts in value integration, such as reduced loss sensitivity, rather than simple increases in arousal. Thus, gambling cues appear to shift the underlying decision policy, consistent with a PIT-like influence of Pavlovian cues on instrumental choice.

Further studies show that gambling cues influence behavior across multiple domains. High-craving gambling images steepen delay discounting and alter striatal value coding without slowing behavior (Miedl et al., 2014), suggesting direct modulation of valuation processes. Simulated slot-machine tasks demonstrate that audiovisual win cues increase subjective engagement and risk taking, with stronger effects in individuals with higher problem-gambling severity, affective symptoms, and in women (Arshad et al., 2025). In healthy volunteers, casino-style cues similarly increase risky choice, reduce attention to probability information, and heighten physiological arousal (Cherkasova et al., 2018; Spetch et al., 2020), paralleling the cue-induced biases observed in animals.

Taken together, animal and human studies show that gambling-related cues bias choice toward risk and increase action invigoration, with strongest effects in phenotypes marked by high incentive salience, impulsivity, or gambling disorder symptoms. Rodent work highlights exaggerated cue responses in sign-tracking and risk-preferring animals, whereas adolescents show little cue-induced risk amplification. Human PIT-like and cue-based tasks similarly reveal shifts in value integration and decision policies, particularly in gambling disorder. Yet because most human paradigms lack a Pavlovian training phase, existing evidence reflects PIT-like motivational capture rather than PIT in the strict sense, underscoring the need for translational PIT designs in clinical gambling populations.

Recent studies on Internet Gaming Disorder (IGD) have begun to implement genuine PIT tasks, allowing a more direct assessment of Pavlovian influences on instrumental behavior. IGD and GD share several features, and contemporary gaming environments increasingly include chance-like reward structures such as loot boxes, consistent with frameworks that conceptualize gaming and gambling along a continuum (Hilbrecht et al., 2020). Across studies, individuals with IGD exhibit enhanced specific PIT for gaming or monetary cues, with PIT magnitude correlating with symptom severity and in some cases predicting gaming time or inflexible responding (Qin et al., 2023; Xu et al., 2024). Longitudinal work further suggests that PIT may reflect a general motivational vulnerability, since PIT responses to non-gaming cues, for example shopping-related stimuli, predicted symptom progression more strongly than gaming cues themselves (Steins-Loeber et al., 2025). These findings indicate that PIT may index broader cue-driven tendencies rather than disorder-specific processes.

### Pavlovian-to-instrumental transfer and Pavlovian-to-decision transfer in binge-eating and obesity

A large preclinical literature has examined how Pavlovian food cues modulate instrumental responding and consummatory behavior, providing insight into motivational processes relevant for binge eating (Hildebrandt and Ahmari, 2021). Although these paradigms were not designed to model binge eating directly, they clarify when food-predictive cues invigorate instrumental behavior, override satiety, or bias choice toward palatable outcomes. One widely used example is cue-potentiated feeding. In this paradigm, presentation of discrete conditioned stimuli (for example auditory or visual cues) previously paired with food can increase consumption in sated rats, and related protocols also use contextual cues as predictors of palatable food availability (Holland and Petrovich, 2005). A context-based variant is that a context previously paired with palatable food leads sated rats to overconsume standard chow. This effect is amplified by small palatable primers and is often used as a relapse-like model of overeating (Boggiano et al., 2009).

PIT studies further differentiate general versus specific motivational influences. When animals are sated on a particular food (sensory-specific satiety), general PIT disappears, but specific PIT remains intact (Lingawi et al., 2022). This dissociation suggests that cues linked to a particular food outcome can continue to bias instrumental behavior even when physiological need is low, whereas general motivational arousal is suppressed. Such persistence of specific PIT is relevant for binge eating and obesity, where cue-driven choice often occurs despite satiety. In line with this obesity-prone rats show stronger specific PIT than obesity-resistant rats despite similar learning, accompanied by altered neural responsivity (Derman and Ferrario, 2018), suggesting that enhanced cue-triggered motivation may increase susceptibility to overeating. Other studies reveal that Pavlovian cues can differentially shape seeking versus consumption, for example when cues predicting the cancellation of food suppress instrumental responding yet increase consumption (Holland, 2014).

Outside the PIT framework, binge-prone rats tolerate higher punishment to obtain palatable food, consume more despite satiety and safer alternatives, and show heightened responding after dieting histories, a pattern often interpreted as an addiction-like drive toward palatable food (Oswald et al., 2011). These findings complement PIT work by demonstrating that motivational drive for palatable food can become resistant to cost and control, mirroring core features of compulsive behavior.

Human PIT studies with food outcomes generally indicate that Pavlovian cues can bias instrumental responding in ways that sometimes compete with current action-outcome contingencies. Across several paradigms, outcome-specific PIT emerges reliably, in a way that cues associated with a particular snack tend to shift choice toward the corresponding action even when that food has been devalued through satiation (Watson et al., 2014). Similar patterns appear when motivational states are altered externally. For instance, anti-sugar videos reduce uncued unhealthy snack seeking, yet cue-elicited responding for those snacks remains unchanged (Kirsten et al., 2021). These patterns mirror animal findings showing that specific PIT can persist under satiety.

Only one study has directly examined PIT in individuals with recurrent binge eating. Chan and Lai (2023) found intact outcome-specific PIT in both groups and no differences between individuals with recurrent binge eating and controls. Cue effects were driven mainly by reduced responding to non-rewarded cues rather than heightened approach to palatable outcomes, suggesting a more context-dependent influence of cues in this paradigm. The authors note that heterogeneity in binge type and the use of non-individualized food cues may have limited the detection of disorder-relevant cue effects.

Beyond binge eating, findings from overweight and obesity samples are mixed. Some studies report no differences in specific PIT by weight status (Meemken and Horstmann, 2019), whereas others find heightened cue sensitivity to high-calorie outcomes (Watson et al., 2017) or even stronger PIT in overweight relative to both normal-weight and obese groups (Lehner et al., 2017). These discrepancies likely reflect differences in cue salience, motivational relevance, or attentional engagement, and eye-tracking work indicates that gaze patterns during Pavlovian learning modulate PIT expression. Importantly, basic instrumental performance and explicit action-outcome knowledge are often preserved in these samples, suggesting that altered cue-driven motivational transfer does not necessarily reflect a global impairment in instrumental control.

Synthesizing these findings, animal models demonstrate that Pavlovian food cues bias instrumental responding primarily through outcome-specific PIT. This effect persists even under satiety and is markedly amplified in vulnerable phenotypes, suggesting that cues shape discrete action selection rather than generating a uniform motivational drive. While this robust animal data provides a clear framework for cue-driven behavior, human findings remain less consistent. Although outcome-specific PIT reliably emerges in human laboratory tasks, evidence for its enhancement in binge eating or obesity is limited and often contradictory. This discrepancy aligns with the view that addiction-like motivational processes may only characterize a specific subset of individuals (Meule et al., 2017). Ultimately, the disconnect between robust animal models and mixed clinical data underscores the necessity for more ecologically valid and targeted PIT designs to isolate the conditions under which cues drive maladaptive eating in humans.

## Discussion

Across paradigms examining cue learning, cue-triggered motivation, and the translation of learned values into behavior, a broadly consistent pattern emerges in alcohol use disorder (AUD), binge-eating-related phenotypes (BED), gambling disorder (GD), and internet gaming disorder (IGD). In these conditions, reward-paired cues can capture attention, invigorate behavior, and bias action selection or decision policies. These effects can occur even when outcomes are devalued, or when costs and probability information would be expected to discourage responding. However, these effects are not consistently observed across tasks, samples, or reinforcer types, and the literature points to pronounced individual differences and boundary conditions rather than a uniform group-level effect.

Mechanistically, the reviewed findings converge on a framework in which repeated cue-reward pairings assign incentive value to predictive stimuli, allowing cues to bias attention and motivational state and, under some conditions, to modulate instrumental control. Crucially, cue influence can be expressed as enhanced motivational pull without necessarily implying impaired learning or deficient explicit knowledge. This dissociation is most clearly illustrated in sign-tracking versus goal-tracking paradigms, where individuals acquire comparable cue-outcome knowledge yet diverge sharply in whether cues become strong motivational magnets (Flagel et al., 2009). Converging human evidence using gaze capture and value-modulated attentional capture similarly suggests graded variation in cue-reactivity, even when overt cue-directed behavior is subtle (Schad et al., 2019; Wardle et al., 2018).

If sign-tracking and attentional capture demonstrate that cues can acquire motivational and attentional priority, PIT provides a direct bridge to action by indexing how Pavlovian value modulates instrumental behavior. PIT is particularly informative because it dissociates cue-driven motivational influence from broader claims about rigid and inflexible control and distinguishes outcome-specific from more general forms of cue-induced invigoration. Evidence from the food domain illustrates this constraint, as sensory-specific satiety attenuates general PIT while leaving outcome-specific PIT intact, indicating that cues can continue to bias choice toward a specific outcome even when global motivational drive is reduced (Lingawi et al., 2022). More broadly, differences between general and specific PIT show that cure-driven effects do not necessarily generalize across reinforcers. However, selective or weak transfer does not imply an absence of cue-control.

Across AUD and GD, PIT and Pavloviantodecision transfer paradigms further show that cue effects depend strongly on task structure and context. In AUD, both outcome-specific and non-drug PIT have been linked to relapse risk and clinical trajectories in some samples (Doñamayor et al., 2021; Garbusow et al., 2016; Sommer et al., 2020), yet the direction and magnitude of transfer vary with abstinence status and task design, including inhibitory cue effects in detoxified individuals (Chen et al., 2022). In GD, most paradigms rely on Pavlovian to decision transfer manipulations rather than classical PIT, for example win-related sensory cues that bias risky choice and attention (Cherkasova et al., 2018). In this domain, cue-driven influence appears less as invigoration of a single learned action and more as a shift in decision policy, such as altered probability weighting or reduced attention to value-relevant information. This interpretation aligns with the broader observation that apparent inconsistencies in gambling and addiction research often reflect differences in task structure and reward type rather than one consistent deficit across studies (Limbrick-Oldfield et al., 2013).

A useful integrative framework for interpreting these converging patterns is incentive sensitization. In its classic formulation, incentive sensitization proposes that repeated reward exposure sensitizes systems that attribute incentive salience, amplifying cue-triggered “wanting” independently of hedonic “liking” (Berridge and Robinson, 1993, 2016). Within the present synthesis, this framework offers a coherent account of why cues become attention-grabbing, persistently motivational, and capable of invigorating instrumental behavior, including after periods of abstinence. It is also compatible with evidence that cue-driven effects can be domain-general at the level of individual differences (Mahler and De Wit, 2010; Morrow et al., 2011). However, the reviewed findings indicate that incentive sensitization should be considered as a candidate shared mechanism rather than a complete explanation, as partially overlapping processes can yield similar behavioral outcomes. Consistent with this view, gambling cues can bias risky choice even in healthy samples, potentially via altered attention to probability information and heightened arousal, mechanisms that align with incentive salience but are not specific to incentive sensitization (Cherkasova et al., 2018). Similarly, food-related paradigms show that outcome-linked cue-control can persist under satiety, as in cue-potentiated feeding or specific PIT, without implying a generalized or progressively sensitized motivational state across individuals (Hildebrandt and Ahmari, 2021; Lingawi et al., 2022).

Across disorders, subgroup differences often provide more insight than mean group differences. In AUD, cue-induced craving and cue-driven behavior vary substantially, and individuals with high cue reactivity show poorer treatment outcomes despite similar baseline severity (Kirsch et al., 2024). These findings suggest that shared clinical phenomena, such as cue-triggered urges or loss of control, do not necessariliy reflect a common mechanism across all individuals within the diagnosis. A transdiagnostic perspective may therefore benefit more from identifying cue-responsive phenotypes that cut across disorders than from attributing heightened cue-driven control to entire diagnostic groups. In this view, disorders such as AUD, BED, GD, and IGD likely include cue-responsive subgroups alongside others whose difficulties reflect different mechanisms.

A major interpretive constraint concerns reinforcer type. Primary and secondary rewards engage overlapping valuation circuitry but differ in their sensory, interoceptive, and representational properties (Sescousse et al., 2013). This distinction matters because many human PIT studies rely on monetary outcomes, whereas animal PIT typically uses primary reinforcers with tightly controlled outcome identities. In addiction and gambling contexts, the abstract and fungible nature of monetary outcomes complicates the interpretation of non-drug PIT as fully neutral with respect to addiction-relevant motivation (Limbrick-Oldfield et al., 2013). If incentive sensitization reflects sensitized attribution of incentive salience, it should in principle apply to both primary and conditioned reinforcers, but its observable behavioral expression may differ depending on whether cues are linked to interoceptive relevance, abstract value, or uncertainty. Reinforcer type therefore constrains how cue-driven effects should be interpreted, not whether cue-driven processes exist.

Variability in human PIT findings, especially across studies of addictive and binge-eating-related disorders, should be interpreted in light of translational limitations. Human PIT tasks vary widely in outcome separation, motivational relevance, and the ability to dissociate specific and general transfer, all of which influence detectability and interpretation. Belanger et al. (2022) argue that inconsistent findings may reflect limitations of current task designs rather than the absence of PIT mechanisms in humans, underscoring the need for improved alignment between animal and human paradigms.

Attentional capture represents one pathway through which cues may bias action by prioritizing cue-related information at the expense of goal-relevant signals such as costs or probability. However, attentional bias alone is neither sufficient nor uniformly associated with cue-driven behavior at the between-person level. While reward learning can produce persistent attentional capture (Anderson and Yantis, 2013), its behavioral impact depends on motivational state and control capacity (Freichel et al., 2024; Garre-Frutos et al., 2025). Evidence from IGD illustrates this interaction, as cue-related attentional capture emerges primarily in individuals with high symptom severity and reduced attentional shifting ability (Heo et al., 2024).

Although this review focused on AUD, BED, and GD, with IGD as a related case, the core pattern likely extends beyond these categories. Evidence for cross-domain cue-reactive phenotypes in humans supports the plausibility of transdiagnostic cue-reactivity traits that are not confined to any single diagnosis (Mahler and De Wit, 2010). Future progress will therefore depend less on demonstrating that cues influence behavior and more on clarifying the mechanisms and conditions that shape cue-driven control. Based on this synthesis, several avenues for future research emerge. First, harmonization between animal and human paradigms is needed, particularly for PIT, with explicit separation of specific and general transfer and alignment of outcome identity and motivational state across samples (Belanger et al., 2022). Second, reward selection should be theory-driven and transparent, as reinforcer type (e.g., disorder specific or unspecific) affects translational interpretability (Limbrick-Oldfield et al., 2013; Sescousse et al., 2013). Third, heterogeneity should be modeled rather than treated as error, for example through subgroup analyses and dimensional approaches that capture variability in cue-reactivity.

A final promising direction is computational modeling. Recent computational accounts formalize craving as a state-dependent amplification of value representations (Konova et al., 2018) and position PIT as a computational bridge through which Pavlovian values bias instrumental behavior (Sebold et al., 2022). These approaches are well suited to reconcile dissociations between subjective craving, cue-reactivity measures, and behavior, and to move the field from descriptive cue effects toward formal, parameter-based accounts of underlying mechanisms.

## Conclusion

The evidence reviewed here supports a coherent synthesis in which cues play an important role in shaping behavior not only in learning what predicts reward, but in shaping attention, motivation, and action selection. Across AUD, BED-related phenotypes, GD, and partially IGD cue-driven influences can bias action selection and decision policies, but these effects are not uniform and often concentrate in identifiable subgroups. The most plausible mechanistic interpretation is therefore neither that all disorders share a single cue mechanism nor that cue effects are too inconsistent to matter, but that partially overlapping cue-driven processes recur across disorders and interact with reinforcer type, task structure, and individual vulnerability.

On this view, cue-driven control represents both a candidate transdiagnostic target and a methodological challenge, as interventions that reduce subjective cue-reactivity do not necessarily diminish cue-control over behavior. Accordingly, progress will require experimental paradigms that directly assess how reward-associated cues influence action and decision-making, such as Pavlovian and Pavlovian-to-Instrumental Transfer paradigms.
